# In times of Zika: getting pregnant or delaying plans

**DOI:** 10.5935/1518-0557.20160051

**Published:** 2016

**Authors:** Viroj Wiwanitkit

**Affiliations:** Surindra Rajabhat University, Thailand

Dear Editor,

The recent report on Zika virus infection is very interesting (Carvalho *et al*., 2016). Carvalho *et al*. (2016) noted that "until now, there is no formal recommendation to avoid pregnancy solely because of the Zika virus outbreak, and the choice of becoming pregnant has been regarded as a personal decision to be made by each woman and her family". Indeed, the concern on reproduction health in the era of Zika virus infection is very important. And the ways of dealing with this challenge might depend on the background in each setting. In my setting, Southeast Asia, the problem already existed but there is still no specific set of recommendations regarding pregnancy planning. Preparing for pregnancy is important, but a more important consideration might be the management of the pregnant population. To provide abortion or not is a big ethical dilemma. Finally, it should be noted that not all infants born from women infected with Zika develop neurological complications. In Asia, most cases are asymptomatic (Wiwanitkit & Wiwanitkit, 2016) and there is no confirmed correlation between Zika virus infection and microcephaly (Wiwanitkit, 2016).
